# Economic Indicators, Quantity and Quality of Health Care Resources Affecting Post-surgical Mortality

**DOI:** 10.1007/s44197-024-00249-x

**Published:** 2024-05-27

**Authors:** Raffaele Merola, Maria Vargas

**Affiliations:** Anesthesia and Intensive Care Medicine, Department of Neurosciences, Reproductive and Odontostomatological Sciences, University of Naples “Federico II”, Naples, Italy

**Keywords:** Anesthesia, Sugery, Post-operative mortality, Health care, Analysis

## Abstract

**Objective:**

to identify correlations between quality and quantity of health care resources, national economic indicators, and postoperative in-hospital mortality as reported in the EUSOS study.

**Methods:**

Different variables were identified from a series of publicly available database. Postoperative in-hospital mortality was identified as reported by EUSOS study. Spearman non-parametric and Coefficients of non-linear regression were calculated.

**Results:**

Quality of health care resources was strongly and negatively correlated to postoperative in-hospital mortality. Quantity of health care resources were negatively and moderately correlated to postoperative in-hospital mortality. National economic indicators were moderately and negatively correlated to postoperative in-hospital mortality. General mortality, as reported by WHO, was positively but very moderately correlated with postoperative in-hospital mortality.

**Conclusions:**

Postoperative in-hospital mortality is strongly determined by quality of health care instead of quantity of health resources and health expenditures. We suggest that improving the quality of health care system might reduce postoperative in-hospital mortality.

## Introduction

Surgery is ubiquity, it occurs in various settings, from the most resource rich to the most resource limited, each with different mortality rates. The demand for surgical procedures has greatly increased with the shifting landscape of disease patterns [1]. Recent estimates suggest that annually, approximately 234.2 million surgical procedures are conducted globally, corresponding to one procedure for every 25 people [1–3]. However, it appears that this number is underreported due to incomplete data and unrecorded interventions. Furthermore, the worldwide distribution of surgeries is unequal: developed countries account for the majority of surgeries, while information on surgeries in developing countries is limited [1].

Post-operative mortality is one of the most universal and measurable outcomes following surgery and consequently surgical death rates are increasingly used as a benchmark to measure hospital quality [4]. Several countries have introduced a successful process of voluntary post-operative mortality audit [5, 6]; however, it remains common for many hospitals to discuss surgical deaths during regular morbidity and mortality conferences. This can lead to underreporting of events and limited impact on improving quality of patient care [7]. Over the past 50 years, post-operative mortality has markedly decreased [8]. Nevertheless, despite all medical and technological advances, post-operative mortality remains above ideal levels. Moreover, significant global disparities imply that standard of evidence concerning perioperative care is insufficient in many countries [9]. In developed countries, surgical procedures were associated to mortality rates of 0.4-0,0.8% while, in developing countries, mortality rates range from 5 to 10% [9]. The European surgical outcomes study (EUSOS), a 7-day cohort study on mortality after surgery across 28 European nations, described the hospital mortality after surgery [10]. In the EUSOS study, the overall in-hospital mortality was 4%, ranging from 1 up to 10% according to different participating countries [10]. Several factors should be considered when ranking countries by post-operative in-hospital mortality such as health care resources, human resources for health, hospital beds, including critical care and intensive care beds, surgical safety, clinical pathways, volume of cases [11].

This study aims to identify correlations between quality and quantity of health care resources, national economic indicators, and postoperative in-hospital mortality as reported in the EUSOS study.

## Methods

Postoperative in-hospital mortality was identified as reported in the EUSOS study by Pearse end colleagues [10]. Quality and quantity of health care resources as well as national economic indicators were identified from publicly available databases from the World Health Organization (WHO), the Organization for Economic Co-Operation and Development (OECD) and the European Commission Database (EUROSTAT) [12–14].

For quality of health care resources, we used the following 5 years observed colorectal cancer survival (HCQICR, Healthcare Quality Indicators for Comparative Reports, Observed) and 5 years relative colorectal cancer survival (HCQICR Relative).

Observed and relative survival as declared by OECD [12] differs in the denominator. For observed survival, the denominator is the number of patients diagnosed with each cancer (age 15–99 years) within a certain period [12]. For relative survival, the denominator is expected survival rate of a comparable group from general population [13].

For quantity of health care resources, we used the following:

- Total density of Magnetic Resonance Imaging (MRI) per million population.
- Total density Positron Emission Tomography (PET) per million population.
- Physicians per 10,000 population.
- Nurses per 10,000 population.
- Hospitals per 100,000 population.
- Hospital beds per 100,000 population.
- ICU (Intensive Care Unit) Beds per 100,000 population.

For national economic indicators we used the following:

- Gross Domestic Product (GDP) per capita in Purchasing Power Standards (PPS).
- Gross national income per capita expressed in PPP.
- Total expenditure for health as percentage (%) of GDP.
- Per capita total expenditure for health expressed in PPP.
- Per capita government expenditure on health expressed in PPP.

For general mortality, we used adult rate as probability of dying between 15 and 60 years of age for 1000 population as defined by WHO [12].

We considered the data declared for the 2011. When the data of 2011 were not available, we have considered eligible the data of 2010.

### Statistical Analysis

Statistical analysis was conducted by using SPSS Software. Spearman non-parametric correlation was used to assess the correlation between variables and postoperative in-hospital mortality. Correlations have been weighted for the total number of patients provided for each country participating to EUSOS study. Correlations were classified before the analysis as mild (rho ≤ 0.4), moderate (0.4 < rho < 0.7) and strong (rho ≥ 0.7) according to rho value [15]. Coefficients of non-linear regression were calculated to assess the effect of each variable on post-surgical mortality. Statistical significance was set at *p* < 0.05.

## Results

### Quality Health Care Resources and Mortality

**Fig. 1:**
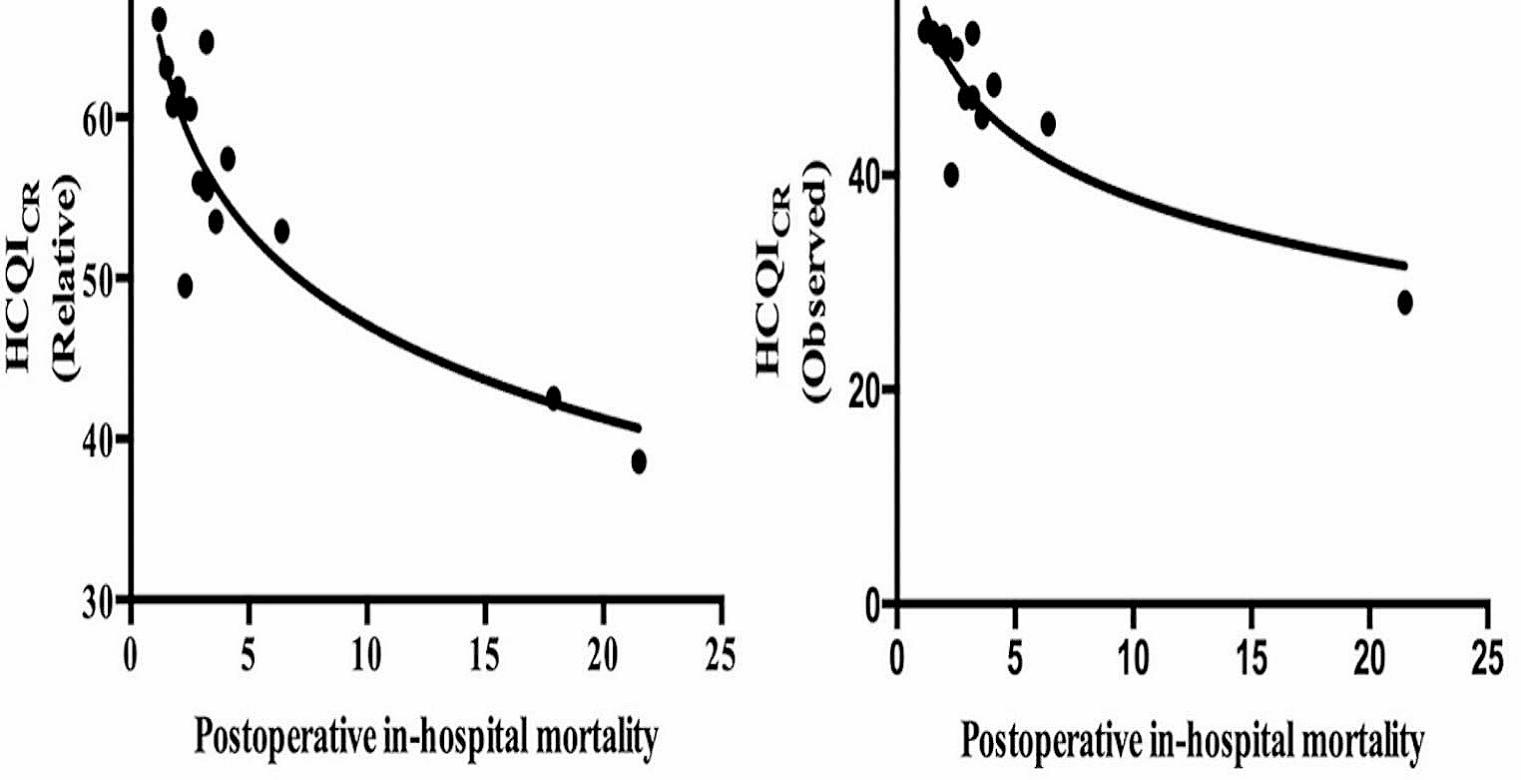
Spearman non-parametric correlation between indicators of healthcare resource quality and postoperative in-hospital mortality. HCQIcr = Health care quality indicator colorectal cancer

Both observed and relative HCQICR showed a strong negative association with postoperative in-hospital mortality (Fig. 1); rho = -0.786; *p* = 0.000; rho = -0.786; *p* = 0.001, respectively (Table 1).

**Table 1:** Results of non-linear regression for postoperative in-hospital mortality and variables included in this study

Variables	B (Beta coefficient)	*p*	Goodness of fit (*r*^2^)
GDP per capita in PPP	-7.575	0.007	0.267
Gross national income per capita	-5.390	0.031	0.180
Total expenditure for health as percentage of GDP	-9.991	0.034	0.168
Per capita total expenditure for health	-4.831	0.006	0.262
Per capita government expenditure on health	-4.571	0.005	0.280
Total density of MRI	-2.207	0.073	0.198
Total density PET	-1.638	0.212	0.167
Physicians	-1.79	0.496	0.022
Nurses	-4.475	0.046	0.227
Hospitals	-2.124	0.397	0.048
Hospital beds	2.607	0.382	0.031
HCQI_CR_ Observed	− 40.369	0.000	0.815
HCQI_CR_ Relative	-42.647	0.000	0.745
General mortality	8.785	0.46	0.150

B = beta coefficient, p = p-value, r2 = goodness of fit

### Quantity Health Care Resources and Mortality

Density of MRI, PET and nurses were moderately and negatively correlated to postoperative in-hospital mortality (MRI: rho = -0.546; *p* = 0,000. PET: rho = -0.583; *p* = 0.000. Nurse: rho = -0.611; *p* = 0.000.). Density of hospital beds, hospital and physicians were mildly and negatively correlated to postoperative in-hospital mortality (Hospital beds: rho = -0.234; *p* = 0,000. Hospital: rho = -0.223; *p* = 0,000. Physicians: rho = -0.077; *p* = 0.000.). Furthermore, ICU beds were very mildly and negatively correlated to postoperative in-hospital mortality (rho = -0.13; *p* = 0.005).

### National Economic Indicators and Mortality

**Fig. 2:**
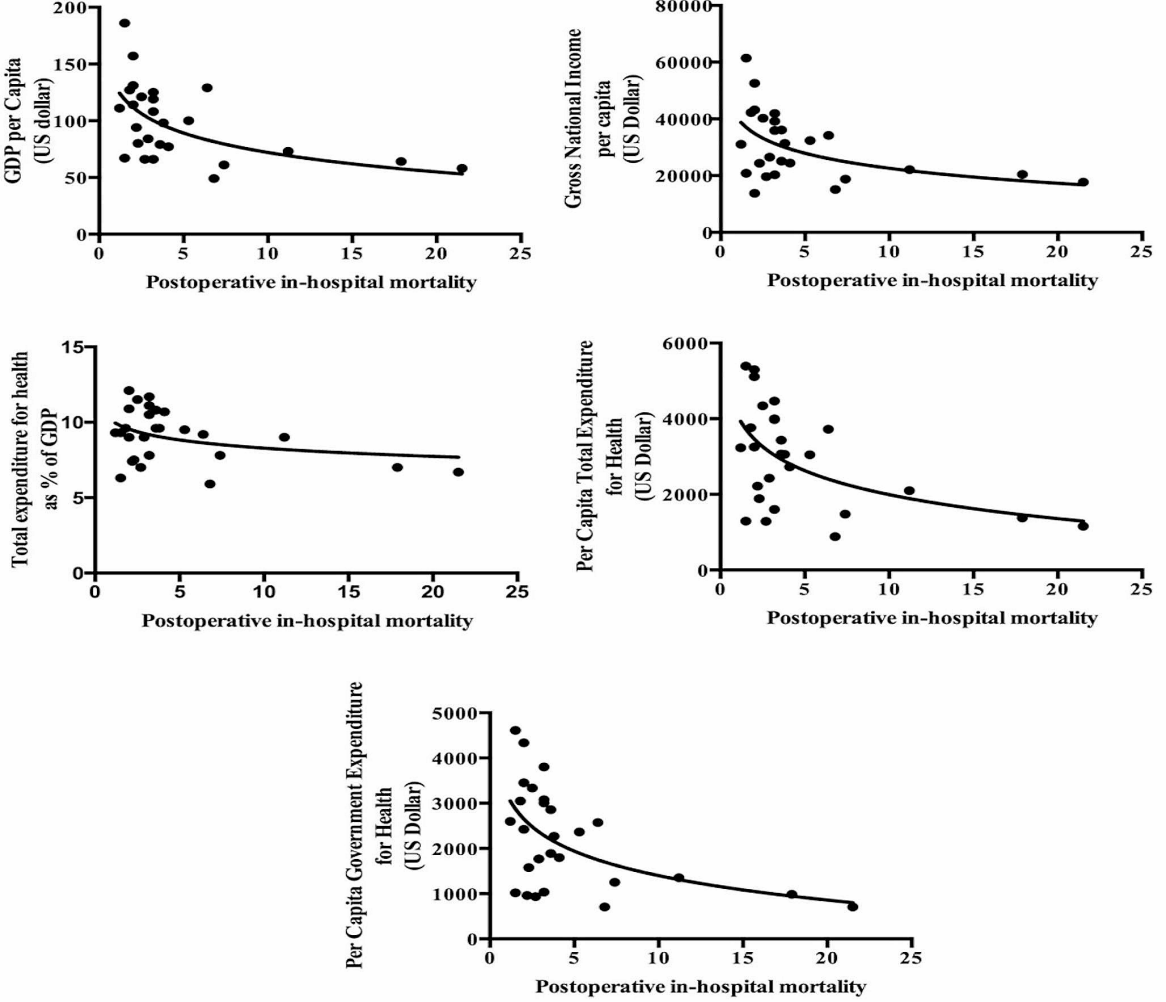
Spearman non-parametric correlation between postoperative in-hospital mortality and national economic indicators

The graphical correlation between national economic indicators and postoperative in-hospital mortality in each country is shown in Fig. 2. National economic indicators were moderately and negatively correlated to postoperative in-hospital mortality (GDP per capita: rho = -0.669, *p* = 0.000; Gross national income: rho = -0.615, *p* = 0.000; Per capita total expenditure for health expressed in PPP: rho = -0.693, *p* = 0.000; Total expenditure for health as percentage (%) of GDP: rho = -0.474, *p* = 0.000).

### General Mortality and Mortality

**Fig. 3:**
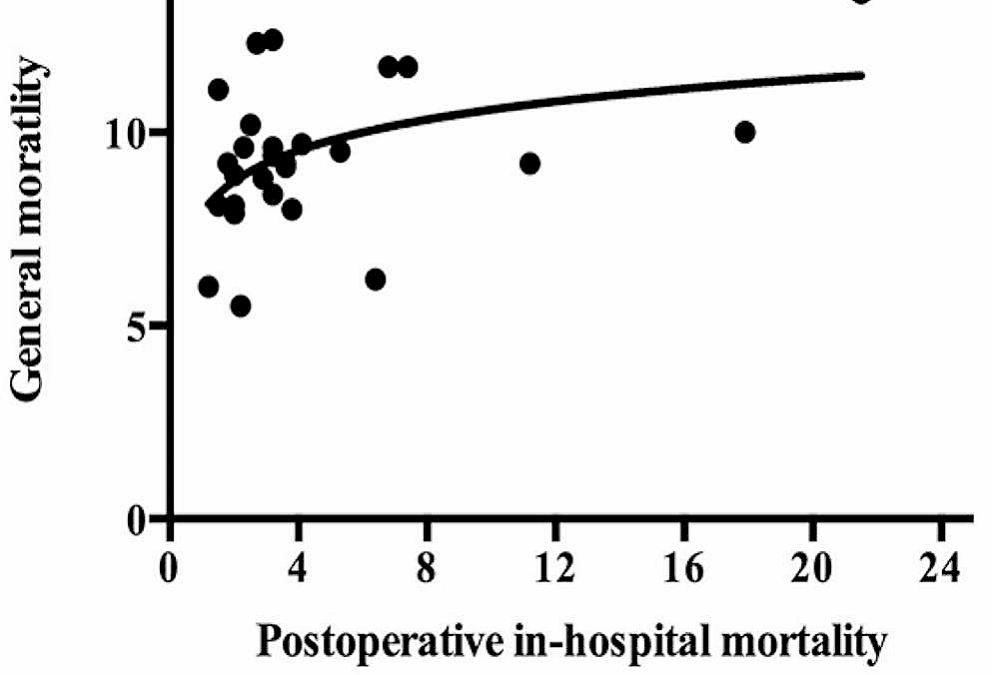
The graphical correlation between postoperative in-hospital mortality and general mortality reported by who

As shown in Fig. 3, general mortality in each country as reported by WHO was positively but very moderately correlated with postoperative in-hospital mortality (rho = 0.401, *p* = 0.038).

### Non-linear Regression

The coefficient, p-value and goodness of fit, of non-linear regression between significantly correlated variables and postoperative in-hospital mortality is shown in Table 1.

Observed and relative HCQICR, as health care quality indicator, had higher impact in determining postoperative in-hospital mortality (Observed HCQICR: B = -40.369; *p* = 0.000 and Relative HCQICR B = -42.647; *p* = 0.000, respectively).

Among quantity of health care resources, only number of nurses/100.000 population were mild predictors of postoperative in-hospital mortality (r2 = 0.227; *p* = 0.046).

Economic indicators were predictors of postoperative in-hospital mortality (all indicators *p* < 0,05). Among them, total expenditure for health as percentage of GDP and GDP per capita in PPS were moderate predictors of postoperative in-hospital mortality (B = -9.991; *p* = 0.0034 and B = -7.575; *p* = 0.007, respectively).

## Discussion

This study investigated the possible correlation between postoperative in-hospital mortality as reported by EUSOS study and quality and quantity of health care resources as well as national economic indicators. The primary findings of our study were: (1) quality of health care resources was strongly correlated with postoperative in-hospital mortality; (2) national economic indicators as well as quantity of health care resources had a moderate to mild correlation with postoperative in-hospital mortality; (3) postoperative in-hospital mortality in the European countries was moderately correlated with general mortality reported by WHO.

Quality of care is that portion of a patient’s outcome over which health care providers, whether individuals or organizations, have control [16]. Quality of health care is determined by different characteristics as the accessibility, effectiveness, efficiency, continuity, efficacy of the care needed [17]. Differences among countries in outcomes may results from differences in the quality of care or in practice patterns driven by socio-economic factors [18].

There are significant difficulties in assessing the relative performance of the health system. These include defining criteria for selecting the mix of indicators to incorporate into the overall measure; the apples and oranges problem of achieving standardization so that benchmarking is calibrated on the same comparative variables; the attribution problem, determining which indicators or variables are more or less influential in determining performance [19]. Performance indicators, defined as measurable elements of practice performance for which there is evidence or consensus that they can be used to assess the quality, and hence change of quality, of care provided, are typically designed to routinely monitor aspects of healthcare performance such as effectiveness, efficiency, safety and quality [20]. The welfare state, life expectancy, adolescent health, natal and maternal mortality have been traditionally considered as indicators of the health care performance and quality [21, 22]. According to our results, there was a strong correlation between postoperative in-hospital mortality and quality of health care. Furthermore, European countries with higher quality of health care system had lower postoperative in-hospital mortality.

Quantity of health care resources as imaging assessment with MRI, CT-scan and PET have a critical positive effect on clinical diagnosis and therapy and provide for comprehensive regional and global structural, functional, and molecular assessment of various clinical disorders [23–25]. Nowadays, imaging techniques have become a necessary tool for diagnosing almost all major types of medical abnormalities and diseases, such as traumatic diseases, many types of cancer diseases, respiratory diseases, cardiovascular diseases, neurological disorders, and many other medical conditions [25].

Imaging is also useful for preoperative evaluation in patients undergoing non-cardiac surgery [26, 27]. Furthermore CT-scan, PET, MRI and any variety of imaging study have been recognized as key determinants for assessing progression-free survival in many clinical trials for solid tumors [28].

Quantity of human resources for health was widely recognized as a crucial determinant of health system performance and of health outcomes [29]. The importance of having adequate human resources for health has been heightened even more during the COVID-19 pandemic [30]. The development and support of human resources for health are essential for achieving better health [31]. Density of physicians and nurse was significantly correlated to the variation in rates of maternal mortality, infant mortality, and under-five mortality across countries [29, 32].

Hospital beds, including critical care and intensive care beds, markedly vary among European countries even if corrected for population size, GDP, expenditure for health care [33, 34]. The seven times greater provision of intensive care beds for Germany than for the UK is likely to influence ICU admission rates and postoperative outcomes [33–35]. This figure is in line with the EUSOS study which shows a higher rate of admission to intensive care after surgery in Germany than in the UK and with other studies showing that less than a third of high-risk non-cardiac surgical patients are admitted to intensive care after surgery in the UK, despite high mortality rates [10, 36–38]. In EUSOS study Pearse et al., suggested that the insufficient number of intensive care beds result in a systematic failure in the ICU resource allocation process and in increased in-hospital mortality. In fact, in the EUSOS study only 5% of patients underwent a planned admission in intensive care with a median stay of about 1 day. Unplanned ICU admissions were associated with higher mortality rates than planned admissions. Surprisingly, most of the patients who died were not admitted to intensive care at any stage after surgery. Of the patients who died after admission to the ICU, nearly half did so after the initial episode was completed and the patient was discharged to a general inpatient ward [10].

In our study, total density of MRI, PET and nurses were moderately correlated to postoperative in-hospital mortality while physicians, density of hospital and hospital beds were mildly correlated to previous mortality. Furthermore, we found a very weak correlation between density of ICU beds and postoperative in-hospital mortality. These findings may suggest that the increase technological resources and staff nurses instead of raising hospital capacity and beds as well as staff physicians might better reduce postoperative in-hospital mortality.

GDP is used to analyze how economies evolve worldwide, while the PPP standardized the differences between national GDP. Lower per capita GDP standardized for PPP were associated with higher proportion of intracerebral hemorrhages, higher incident risk of stroke and mortality [39, 40]. It has been also reported an inverse correlation between economical indices, like GDP with national income and infant mortality ratio [41]. GDP and national income levels were also negatively and inversely correlated with burn mortality, since this kind of mortality lead to prolonged, expensive and complex hospital stay [42]. Furthermore, health care expenditure and total public spending rise with national income, this makes expenditure on health particularly important in poor countries [43]. Health care expenditure was associated with the growth of GDP [44]. Private and government health spending per capita had a significant effect on reducing infant, child and maternal mortality [45, 46]. In our study, GDP, national income and different expenditures for health had a moderate and negative correlation with to postoperative in hospital mortality. According to our results, not only the national wealth, but the also the different percentages of it spent for health may affect postoperative in hospital mortality.

The EUSOS study reported an overall in-hospital mortality of 4% for surgical procedures [10]. The mortality rate of different countries in EUSOS analysis aroused many discussions due to fact that mortality was over-reported in some centers [47–51]. In the present study, the mortality rate of each country reported by EUSOS study was compared with the death rate publicly available in WHO registry. We found that postoperative in-hospital mortality had moderate and positive correlation with general mortality.

The relationship between health expenditure and infant as well as child mortality was investigated by Farag et al., using data from 133 low and middle income countries [45]. They found that GDP per capita standardized for PPP and total health spending as percentage of GDP were the greater determinants of infant and child mortality (B = -0,5845, B = -0.3275; B = -0.6405, B = -0.3767; respectively) [45]. In United States, per capita expenditure on public welfare is associated with lower working age mortality for both sexes. This implies that if spending on public welfare comes closer to meeting an estimate of need, it is more likely to be associated with lower mortality [52]. In our regression model, HCQI was the best determinant of postoperative in-hospital mortality.

### Limitations

This study has limitations that need to be addressed. Data of postoperative in-hospital mortality refer only to the European countries participating to EUSOS study, therefore they cannot be considered universal, given the lack of data from regions and countries other than those analyzed. Data describing economic indicators, quantity and quality of health care resource, identified from publicly available database, have been included in the analysis if declared at least 50% of the countries participating to EUSOS study. Health care quality indicators have been chosen according to OECD forum on quality of care [53]. Excluding the outliers of postoperative in-hospital mortality from the statistical analysis, the correlation maintains strong for quality of health care resources but became weak for national economic indicator. The definition of ICU beds might differ among countries limiting the analysis and interpretation [33–35].

## Conclusions

Postoperative in-hospital mortality in European countires is strongly determined by the quality of healthcare rather than the quantity of healthcare resources and healthcare expenditure. We suggest that improving the quality of healthcare system performance could improve clinical outcomes and thus reduce postoperative in-hospital mortality. Finally, we would like to emphasize that in order to make the results of this study universal, future research needs to produce data regarding postsurgical mortality in other regions.

## Data Availability

No datasets were generated or analysed during the current study.
